# The impact of human papillomavirus 16/18 positivity on cyberchondria levels in women undergoing colposcopy

**DOI:** 10.1590/1806-9282.20241692

**Published:** 2025-06-16

**Authors:** Hande Nur Öncü, Gökçen Ege, Serap Topkara Sucu, Neslihan Öztürk, Candost Hanedan, Vakkas Korkmaz

**Affiliations:** 1 University of Health Sciences, Ankara Etlik City Hospital, Faculty of Medicine, Department of Gynecologic Oncology – Ankara, Turkey.; 2 University of Health Sciences, Ankara Etlik City Hospital, Faculty of Medicine, Department of Obstetrics and Gynecology – Ankara, Turkey.

**Keywords:** Information seeking behavior, Human papillomavirus, Colposcopy

## Abstract

**OBJECTIVE:**

The negative consequences of the widespread use of the Internet for health-related information have recently become a topic of research. Recently, the impact of internet information-seeking on anxiety has been defined as cyberchondria, and a standardized scoring system has been developed to document it. However, the significance of this scoring system in gynecologic oncology has not yet been established. Therefore, the aim of this study was to evaluate the impact of human papillomavirus 16/18 positivity on the level of cyberchondria in women with human papillomavirus positivity undergoing colposcopy.

**METHODS:**

The study included 333 women with human papillomavirus 16/18 and other high-risk human papillomavirus positivity who underwent colposcopy in the gynecologic oncology clinic. Participants were divided into two groups: human papillomavirus 16/18 positive (Group 1, n=201) and other high-risk human papillomavirus positive (Group 2, n=132). Both groups were administered the short form of the Cyberchondria Severity Scale (CSS-12). Demographic and clinical findings and cyberchondria scores were compared between the groups.

**RESULTS:**

The median ages of the study groups were 36 (26–59) and 40 (25–63) years, respectively (p=0.004). Both groups had similar body mass index, parity, menopausal status, and educational levels (p>0.05). When comparing the four subscales and the total score, the excessiveness (11 [3–15] vs. 9 [3–15], p<0.001, respectively) and the total CSS-12 score (31 [12–55] vs. 28 [12–49], p=0.002, respectively) were statistically significantly different between the two groups.

**CONCLUSIONS:**

In women with human papillomavirus 16/18 positivity undergoing colposcopy, cyberchondria levels were found to be significantly higher. Specifically, excessive information-seeking behavior significantly contributed to this elevated level of cyberchondria.

## INTRODUCTION

Cyberchondria is defined as increased anxiety and worry ­resulting from excessive or repetitive online searches for ­medical information about one's health^1^. The concept has gained prominence with the advent of technology and the easy accessibility of information. In 2017, it was reported that 74.4% of the people in the United States searched for health information online^2^. In 2014, McElroy and Shevlin developed the Cyberchondria Severity Scale (CSS), consisting of 33 items^3^. Subsequently, aiming to assess with fewer items while maintaining the ­validity and reliability of the original scale, a 12-item short form (CSS-12) was published^4^.

Cervical cancer is the fourth most common cancer among women globally, and despite screening and vaccination programs, it remains a significant cause of morbidity and ­mortality^5^. Human papillomavirus (HPV) is responsible for 99.7% of cervical cancers. Of the more than 100 types of HPV, 14 types (16, 18, 31, 33, 35, 39, 45, 51, 52, 56, 58, 59, 66, and 68) are classified as high-risk HPV (hr-HPV). The American Society of Colposcopy and Cervical Pathology (ASCCP) recommends cervical cancer screening for women aged 25–65 years with HPV-based testing every 5 years or co-testing with cytology. If HPV testing is not available, screening with cytology alone should begin at the age of 21 years and be conducted every 3 years. HPV 16 and HPV 18 are responsible for approximately 70% of cervical cancer cases and are direct indications for colposcopy. Hr-HPV types other than HPV 16 and HPV 18 are classified as "other hr-HPV." Colposcopy is indicated when these types are accompanied by abnormal cytology^6^.

Women who test positive for HPV often experience ­anxiety, shame, sadness, and concern, particularly when they learn about the virus's sexually transmitted nature and its association with cervical cancer^7^. The anxiety may be exacerbated by the unfamiliarity of the colposcopy procedure. In societies where women's health issues are not openly discussed, women may turn to the Internet to research their conditions. Given that HPV 16 and HPV 18 cause the majority of cervical cancers, it may lead to greater anxiety among patients undergoing colposcopy. In this study, we aimed to compare the levels of cyberchondria between women who tested positive for HPV 16/18 and those who tested positive for other hr-HPV types and underwent colposcopy.

## METHODS

This prospective observational study was conducted between May 2024 and September 2024 in the gynecologic oncology clinic, after receiving approval from the local ethics committee (approval no. AEŞH-BADEK-2024-398). A total of 333 women scheduled for colposcopy were included in the study. Participants were divided into two groups: HPV 16/18 ­positive (Group 1, n=201) and other hr-HPV positive (Group 2, n=132). Exclusion criteria included the following: lack of internet use, absence of HPV positivity, prior colposcopy, history of psychiatric disorders, use of psychiatric medication, or refusal to participate.

Colposcopy indications and procedures in our clinic adhere to the ASCCP guidelines^6^. HPV results are reported as HPV 16, HPV 18, or other HPV types. In our clinic, HPV types other than HPV 16/18 are classified as "other HPV."

To assess cyberchondria levels, the 12-item CSS was ­administered face to face by trained researchers approximately 15 min before the colposcopy procedure. Participants were informed about the study objectives and provided written informed consent.

The CSS-12 is a validated 5-point Likert scale (1: never, 2: rarely, 3: sometimes, 4: often, 5: always) consisting of four subscales:

- **Excessiveness:** Excessive or repetitive searching for medical information online;
- **Compulsion:** Disruptions in daily life caused by intensive online searches;
- **Distress:** Psychological symptoms, such as insomnia and depression, triggered by negative online search results;
- **Reassurance:** Seeking reassurance from healthcare professionals or other sources.

The total cyberchondria score is calculated by summing the scores from all 12 items, with higher scores indicating greater levels of cyberchondria.

Demographic and clinical data, including age, body mass index (BMI), parity, menopausal status, education level, marital status, cytology results, and HPV status, were extracted from medical records and compared between the two groups. Additionally, the CSS-12 total score and its four subscales (excessiveness, compulsion, distress, and reassurance) were analyzed to assess differences in cyberchondria levels between the groups.

### Statistical analysis

IBM SPSS Statistics for Mac version 22.0 (Armonk, New York, USA) was used for statistical analyses. Descriptive statistical tests were employed to determine the median (±range), minimum, and maximum values for continuous patient data. Categorical variables were expressed as numbers and ­percentage values. The chi-square test was used for categorical data in pairwise ­comparisons, and the Mann-Whitney U test was used for ­continuous data due to their non-parametric distribution. A p-value of ≤0.05 was considered statistically significant.

## RESULTS

The median age was 36 (26–59) years in Group 1 and 40 (25–63) years in Group 2 (p=0.004). In Group 1, 13.4% (27/201) of the patients were menopausal, compared to 15.9% (21/132) in Group 2 (p=0.258). The BMI, marital status, ­parity, and education level were similar between the two groups (p≥0.05). The sociodemographic data of the participants are shown in Table 1.

**Table 1:** Patient characteristics.

Variables		Group 1 (n=201)	Group 2 (n=132)	p-value
Age (years)		36 (26–59)	40 (25–63)	**0.004**
BMI (kg/m^2^)		28 (20–41)	28 (22–36)	0.187
Parity		2 (0–4)	2 (0–4)	0.258
Menopause status (n, %)				0.529
	Yes	27 (13.4%)	21 (15.9%)	
	No	174 (86.7%)	111 (84.1%)	
Marital status (n, %)				0.297
	Married	135 (67.2%)	90 (68.2%)	
	Single	32 (15.9%)	14 (10.6%)	
	Divorced	34 (16.9%)	28 (21.2%)	
Education level (n, %)				0.917
	Elementary school	30 (14.9%)	21 (15.9%)	
	Middle school	31 (15.4%)	17 (12.9%)	
	High school	77 (38.4%)	50 (37.9%)	
	Higher school	63 (31.3%)	44 (33.3%)	

Data are expressed as median, minimum, and maximum. p≤0.05 significant difference, comparison of groups. BMI: body mass index. Statistically significant values are denoted in bold.

The cytology results of the groups were evaluated. In Group 1, 28.9% (58/201) of the women had atypical squamous cells of undetermined significance (ASCUS), 1% (2/201) had atypical squamous cells, cannot exclude high-grade squamous intraepithelial lesion (ASC-H), 7% (14/201) had low-grade squamous intraepithelial lesion (LSIL), 2.4% (5/201) had high-grade squamous intraepithelial lesion (HSIL), 31.8% (64/201) were negative, and 28.9% (58/201) had unknown results. In Group 2, 68.9% (91/132) of the women had ASCUS, 1.5% (2/132) had ASC-H, and 29.6% (39/132) had LSIL. The cytology results for both groups are presented in Table 2.

**Table 2:** Cytology information of the women in the study groups.

Cytology	Group 1 (n=201)	Group 2 (n=132)
ASCUS (n, %)	58 (28.9%)	91 (68.9%)
ASC-H (n, %)	2 (1%)	2 (1.5%)
LSIL (n, %)	14 (7%)	39 (29.6%)
HSIL (n, %)	5 (2.4%)	0
Negative (n, %)	64 (31.8%)	0
Unknown (n, %)	58 (28.9%)	0

ASCUS: atypical squamous cells of undetermined significance; ASC-H: atypical squamous cells, cannot exclude high-grade squamous ıntraepithelial

The four subscales and total cyberchondria levels of the participants were also analyzed. The comparisons between Group 1 and Group 2 were as follows: compulsion (7 [3–15] vs. 7 [3–15], p=0.056), excessiveness (11 [3–15] vs. 9 [3–15], p<0.001), distress (4 [3–12] vs. 4 [3–11], p=0.110), reassurance (8 [3–15] vs. 8 [3–15], p=0.058), and the total CSS-12 score (31 [12–55] vs. 28 [12–49], p=0.002). The CSS-12 score comparisons between the two groups are provided in Table 3.

**Table 3:** The level of cyberchondria between the two groups, including subgroups.

Variables	Group 1 (n=201)	Group 2 (n=132)	p-value
Excessiveness	11 (3–15)	9 (3–15)	**<0.001**
Compulsions	7 (3–15)	7 (3–15)	0.056
Distress	4 (3–12)	4 (3–11)	0.11
Reassurance	8 (3–15)	8 (3–15)	0.058
Total score	31 (12–55)	28 (12–49)	**0.002**

Data are expressed as median, minimum, and maximum. p≤0.05 significant difference, comparison of groups. Statistically significant values are denoted in bold.

## DISCUSSION

To our knowledge, this study is the first to evaluate the impact of HPV 16/18 on cyberchondria in the literature. Using the CSS-12, we found that women who were HPV 16/18 positive had significantly higher levels of cyberchondria compared to those with other hr-HPV types. We also found that this group exhibited substantially higher excessive or repetitive online ­medical information-searching behaviors. These results ­suggest that while the Internet offers benefits such as easy access, anonymity, affordability, and a wide range of information, it can also lead to increased health anxiety due to confusing, ­conflicting, incorrect, and incomplete information.

The widespread use of HPV-based screening has been the subject of numerous studies examining the levels of anxiety it induces in women. McCaffery et al.^7^ found that while HPV screening benefits cervical cancer screening programs, it also leads to adverse social and psychological outcomes for women, mainly due to the sexually transmitted nature of the virus and its association with cervical cancer. Women reported feeling stigmatized, anxious, and stressed, expressing concerns about their sexual relationships and the fear of disclosing their results to others. Women, regardless of their level of development, are also concerned about going to healthcare centers for cervical cancer screening^8^. Therefore, self-HPV tests have been introduced. Lorenzi et al.^9^ found that self-collected hr HPV tests, when combined with biomarkers, have the same level of accuracy as those collected by healthcare professionals. In another study, they evaluated self-sampling by age and found that younger women experienced more fear and discomfort with self-sampling, but regardless of age, it was considered easy to understand and accept^10^. In a 2011 study conducted in Taiwan, the psychological responses of 20 women who learned they were HPV-positive were evaluated. The study found that most women searched the Internet for information about HPV and reputable doctors after learning about their infection, and many reported experiencing negative emotions, such as fear, anxiety, and doubt^11^. However, while these studies show that HPV increases anxiety levels, none have focused on the effects of the most common hr-HPV types, HPV 16/18.

Alay et al.^12^ assessed the sexual function and anxiety levels of patients with HPV 16/18 (normal/abnormal cytology) and other hr-HPV (normal/abnormal cytology) by using the Female Sexual Function Index (FSFI) and the Beck Anxiety Inventory (BAI) at 2-month intervals. They found that women positive for HPV 16/18 had significantly higher BAI scores compared to those positive for other hr-HPV types. The study also indicated that a diagnosis of HPV 16/18 negatively impacts ­women's ­sexual lives. In a similar study conducted in 2023 using the FSFI and BAI indices, it was demonstrated that women with HPV 16/18 positivity had higher levels of anxiety compared to women with other hr-HPV types^13^. In another study conducted on 178 Portuguese women infected with HPV, it was shown that HPV 16/18 positivity is significantly associated with a lower quality of life compared to other HPV types^14^. Although these studies have focused on the impact of HPV 16/18 on ­anxiety, none have demonstrated its effect on cyberchondria levels. Turhan Cakir's study, which evaluated the relationship between cyberchondria and HPV, compared the levels of cyberchondria in women with abnormal uterine bleeding (AUB) and HPV positivity. The study found that the group with HPV positivity had significantly higher levels of cyberchondria (67.43±19.87 vs. 84.16±21.08, respectively)^15^. These findings are consistent with our study; however, our study distinguishes itself by specifically demonstrating the impact of HPV 16/18. Therefore, we believe that our study will contribute to the literature as it is the first study to evaluate the level of cyberchondria in women with HPV 16/18 positivity.

Women with abnormal screening results who are referred for colposcopy experience high levels of stress and anxiety. Marteau et al.^16^ identified that the main concerns of women were whether the colposcopy would be painful and what would happen during the procedure. Baser et al.^17^ evaluated the State-Trait Anxiety Inventory (STAI) scores before the colposcopy and the visual analog scale (VAS) scores after the procedure. They found that women with high levels of anxiety before the procedure had significantly higher pain scores after the procedure. Therefore, many studies have been conducted in an attempt to find ways to reduce this anxiety in women undergoing colposcopy. In Kincey et al.'s^18^ study, 28 out of 30 women believed that a brochure provided before the appointment would help reduce anxiety associated with colposcopy. Another study demonstrated that playing music during colposcopy, ­showing informational videos before the procedure, and watching ­videos during the procedure helped reduce anxiety^19^. Although these studies have shown that providing information can reduce anxiety levels, our study indicates that excessive online health information searching may increase anxiety levels. We believe that the high level of excessiveness, or excessive and repetitive information seeking, among women positive for HPV 16/18 is due to their association with the HPV types most strongly related to cervical cancer.

In conclusion, HPV 16/18 positivity, recognized as a significant contributor to cervical cancer worldwide, is associated with higher levels of cyberchondria in women undergoing colposcopy. This increase is predominantly driven by excessive information-seeking behaviors. To mitigate the potential adverse effects on women's well-being, healthcare professionals should approach discussions about HPV results with ­sensitivity and ensure that the information provided is clear, accurate, and tailored to the patient's needs.
